# Correlation analysis and recurrence evaluation system for patients with recurrent hepatolithiasis: a multicentre retrospective study

**DOI:** 10.3389/fdgth.2024.1510674

**Published:** 2024-11-27

**Authors:** Zihan Li, Yibo Zhang, Zixiang Chen, Jiangming Chen, Hui Hou, Cheng Wang, Zheng Lu, Xiaoming Wang, Xiaoping Geng, Fubao Liu

**Affiliations:** ^1^ Department of General Surgery, The First Affiliated Hospital of Anhui Medical University, Hefei, China; ^2^ Cardiology Division, Department of Medicine, Li Ka Shing Faculty of Medicine, The University of Hong Kong, Hong Kong, Hong Kong SAR, China; ^3^ Department of Analytics, Marketing and Operations, Imperial College London, London, United Kingdom; ^4^ Department of General Surgery, The Second Affiliated Hospital of Anhui Medical University, Hefei, China; ^5^ Department of General Surgery, The First Affiliated Hospital of the University of Science and Technology of China, Hefei, China; ^6^ Department of General Surgery, The First Affiliated Hospital of Bengbu Medical College, Bengbu, China; ^7^ Department of General Surgery, The First Affiliated Hospital of Wannan Medical College, Wuhu, China

**Keywords:** recurrent hepatolithiasis, machine learning, prediction model, high-order correlation data, machine learning operations

## Abstract

**Background:**

Methods for accurately predicting the prognosis of patients with recurrent hepatolithiasis (RH) after biliary surgery are lacking. This study aimed to develop a model that dynamically predicts the risk of hepatolithiasis recurrence using a machine-learning (ML) approach based on multiple clinical high-order correlation data.

**Materials and methods:**

Data from patients with RH who underwent surgery at five centres between January 2015 and December 2020 were collected and divided into training and testing sets. Nine predictive models, which we named the Correlation Analysis and Recurrence Evaluation System (CARES), were developed and compared using machine learning (ML) methods to predict the patients’ dynamic recurrence risk within 5 post-operative years. We adopted a k-fold cross validation with k = 10 and tested model performance on a separate testing set. The area under the receiver operating characteristic curve was used to evaluate the performance of the models, and the significance and direction of each predictive variable were interpreted and justified based on Shapley Additive Explanations.

**Results:**

Models based on ML methods outperformed those based on traditional regression analysis in predicting the recurrent risk of patients with RH, with Extreme Gradient Boosting (XGBoost) and Light Gradient Boosting Machine (LightGBM) showing the best performance, both yielding an AUC (Area Under the receiver operating characteristic Curve) of∼0.9 or higher at predictions. These models were proved to have even better performance on testing sets than in a 10-fold cross validation, indicating that the model was not overfitted. The SHAP method revealed that immediate stone clearance, final stone clearance, number of previous surgeries, and preoperative CA19-9 index were the most important predictors of recurrence after reoperation in RH patients. An online version of the CARES model was implemented.

**Conclusion:**

The CARES model was firstly developed based on ML methods and further encapsulated into an online version for predicting the recurrence of patients with RH after hepatectomy, which can guide clinical decision-making and personalised postoperative surveillance.

## 1 Introduction

### 1.1 Background

Hepatolithiasis is a benign disease that is common in Asia, including China, Japan, and South Korea, with a prevalence of 20%–50% (1, 2). In recent years, the prevalence of this disease has been increasing in Western countries, probably due to increased immigration from the East and changes in Western dietary habits (3, 4). Although benign, hepatolithiasis is a disease that is difficult to treat and, thus, characterised by high rates of treatment failure and recurrence. It can lead to progressive biliary strictures, liver abscesses, cirrhosis, liver atrophy, and even cholangiocarcinoma (5).

Hepatolithiasis is treated with medications and non-surgical methods, such as endoscopy, as well as with surgical procedures (6). As non-surgical methods have various limitations, hepatectomy has better generalisability, lower rates of residual stones, and lower recurrence rates (7). According to the available studies, hepatectomy for hepatolithiasis is associated with a higher survival rate and lower incidences of bile duct stenosis, recurrence, and cholangitis (8).

Recurrent hepatolithiasis (RH) is the recurrence of hepatolithiasis in patients who have undergone medical treatments for hepatolithiasis, such as partial hepatectomy, choledochotomy, and lithotripsy. RH is difficult to resolve because of stone re-formation and pyogenic cholangitis (9, 10). Therefore, effective prediction of patient prognosis is of great significance in guiding decision-making and personalised postoperative surveillance.

### 1.2 Rationale and knowledge gap

According to our previous studies, the Nakayama classification (based on stone distribution), the classification proposed by Tsunoda et al. (based on dilatation or stenosis), the Chinese classification model proposed by the Biliary Tract Research Group of the Chinese Medical Association, and a nomogram based on traditional linear regression have some value in predicting the prognosis of patients with RH (11). However, these methods use linear assumptions and cannot simulate complex, multidimensional, and non-linear relationships between different predictor variables in biological systems; thus, their predictive performance is limited. They are also extremely complex and expensive to learn, and the inability to obtain information about risk changes in the postoperative period and intuitive predictions renders it difficult to use for clinical guidance. Novel solutions capable of handling potentially non-linear variables are in high demand for accurate predictions.

### 1.3 Objective

Machine learning (ML) is a field of artificial intelligence (AI) that can uncover differences and connections in complex and large datasets and can be used to predict future outcomes (12). Hence, we aimed to apply an ML approach, named the Correlation Analysis and Recurrence Evaluation System (CARES), to build a recurrence risk prediction model for RH patients after surgery using nine ML models, based on a multicentre database.

This manuscript is written following STROBE checklist.

## 2 Materials and methods

### 2.1 Study population

The clinical and prognostic data of 1,962 patients who underwent surgery for hepatolithiasis between January 2015 and December 2020 at the First Affiliated Hospital of Anhui Medical University, Second Affiliated Hospital of Anhui Medical University, First Affiliated Hospital of the University of Science and Technology of China, First Affiliated Hospital of Bengbu Medical College, and First Affiliated Hospital of Wannan Medical College were retrospectively collected. All five regional medical centres are tertiary hospitals and high-volume surgical centres that use similar approaches to treat hepatolithiasis. Standardized treatment of patients can provide greater benefits while minimizing risks such as misdiagnosis and underdiagnosis. In addition, it helps to eliminate bias due to inconsistent treatment strategies or assessment criteria.

### 2.2 Ethics approval

The study was conducted in accordance with the Declaration of Helsinki (as revised in 2013). The study was approved by institutional ethics committee of the First Affiliated Hospital of Anhui Medical University (NO. Quick-PJ2021-08-19), and the need for obtaining informed consent was exempted owing to the retrospective nature of the present study.

### 2.3 Inclusion and exclusion criteria

The inclusion criteria were as follows: (1). having undergone at least one biliary surgery for hepatolithiasis; (2). preoperative imaging confirming RH; (3). intraoperative confirmation of hepatolithiasis; (4). preoperative Child-Pugh classification of grade A or B that improved to grade A. The exclusion criteria were as follows: (1). history of abdominal surgery not involving the biliary system; (2). combined with malignancy; (3). incomplete clinical or follow-up data; (4). perioperative death.

### 2.4 Data collection

#### 2.4.1 Preoperative examination and preparation

Basic patient information, including age, sex, body mass index, time of previous surgery, surgical procedure, and symptoms before admission, was retrospectively collected. Preoperative blood markers, including liver and renal function, blood counts, tumour markers, and coagulation factors, were collected at least 1 week before surgery. Inflammation-based scores were calculated, including the albumin/globulin, neutrophil/lymphocyte, and platelet/lymphocyte ratios. Imaging tools, including ultrasound (US), computed tomography (CT), magnetic resonance imaging, and magnetic resonance cholangiopancreatography (MRCP), were selectedly used to document in detail the distribution of stone locations, biliary narrowing, and hepatic lobe atrophy. In some patients with complex bilateral stones, the future residual liver volume and total functional liver volume were calculated using three-dimensional visualisation techniques, and the indocyanine green 15 min retention rate was tested to ensure the safety of the procedure. This test will not be used in patients with a history of indocyanine green allergy and a history of iodine allergy (indocyanine green contains iodine and therefore may cause iodine allergy). If the patients did not reach Child-Pugh class A preoperatively, they received hepatoprotective therapy until their liver function improved to Child-Pugh class A.

#### 2.4.2 Intraoperative strategy and findings

All the surgeries were performed by experienced hepatobiliary surgeons. As patients who had undergone one or more laparotomies tended to have more severe abdominal adhesions, a detailed surgical plan and biliary drainage strategy were formulated based on the location of the stone, sphincter of Oddi function, cirrhosis, and hepatic lobe atrophy, which were confirmed in the preoperative examination and reconfirmed intraoperatively after the surgery. Detailed intraoperative findings, operative approach and duration of surgery were recorded, and choledochoscopy was performed to assess whether the stones were immediately removed. Bile acid was collected intraoperatively for bacterial culture and drug sensitivity testing.

#### 2.4.3 Postoperative examination examination and decision

Postoperative specimens were pathologically diagnosed and described by experienced pathologists from five medical centres. Postoperative complications, including bile leakage, pancreatic fistula, infection, and abdominal bleeding, as well as postoperative blood markers, bile culture, and blood culture results were recorded. Before discharge, abdominal CT and cholangiography or choledochoscopy was used in patients with external T-tube drainage to confirm whether the stone was immediately removed. For patients without instant clearance, choledochoscopy is usually performed through the T-tube sinus tract several times at 6–8 weeks postoperatively until the stone is removed or cannot be removed by any means. For patients with instant clearance, T-tube cholangiography was performed 2 weeks postoperatively. If residual stones were observed, choledochoscopy would be performed, as described above.

#### 2.4.4 Follow-up and data collection

All patients were followed up every 3 months after discharge by the supervising physician in the hepatobiliary surgery clinic or by telephone. Follow-up evaluation included assessment of clinical signs and symptoms, routine blood tests, liver function assessment, and US, CT, or MRCP for residual or recurrent stones. Prognosis was evaluated according to the Terblanche criteria (13) and was considered poor if it was Terblanche classification grade III (serious bile duct-related symptoms requiring treatment) or IV (with anastomotic stricture or bile duct stone formation requiring surgical treatment, resulting in disease-related cancer or death), which was the endpoint of this study.

#### 2.4.5 Missing data handling

Regarding data collection, missing data were dealt with differently in model training and deployment.

During Model Training, for the construction of our machine learning model, we believe in utilizing the most complete and accurate dataset possible. Thus, when an entry has one or more missing feature values, we decided to exclude it from the training process. This approach ensures that our model is trained only on complete cases, minimizing potential biases or inaccuracies that might arise from imputed data.

In our preprocessing steps, the dropna() function was employed to exclude such entries. We're confident that this method is appropriate given our dataset's size and the relative infrequency of missing values. Moreover, we ensured that the removal of these data points did not introduce any bias by examining the distribution of outcomes among the dropped and retained entries.

During Model Deployment, we deemed that in a real-world clinical setting, excluding a patient's data due to a single missing value might not be feasible or desirable. Thus, when our model is used on new patient data, if any feature values are missing, we replace them with the average (mean) value derived from our training dataset. It allows our model to generate predictions even when some data might be temporarily unavailable or missing, and using the mean value from our training set serves as a neutral placeholder, minimizing the potential impact on the model's prediction.

### 2.5 Statistical analysis

#### 2.5.1 Data splitting

In our study, the dataset was divided between training and testing sets. The patient data from the First Affiliated Hospital of Anhui Medical University, Second Affiliated Hospital of Anhui Medical University, and First Affiliated Hospital of the University of Science and Technology of China (82.7%) were used for the training set and those from the First Affiliated Hospital of Bengbu Medical College and First Affiliated Hospital of Wannan Medical College (17.3%) for the testing set. This testing set is entirely independent from the training set, thereby enabling out-of-sample evaluation.

Differences in the clinical characteristics of the included patients were compared using independent samples *t*-test, Mann–Whitney *U*-test, or *χ*^2^ test, and the statistical significance level was set at 0.05.

#### 2.5.2 Model training

Nine machine learning models were used to build a predictive model for recurrence after RH. These models were selected because they represent different types of machine learning algorithms, including linear models [Logistic Regression (LR)], tree-based models [Decision Tree (DT), Random Rorest (RF), Light Gradient-Boosting Machine (LightGBM), Extreme Gradient Boosting (XGBoost)], integrated methods [XGBoost and Adaptive Boosting (AdaBoost)], support vector machine (SVM), neural network (NNW), and instance-based methods [K-nearest neighbour (KNN)]. By comparing the performance of these different models, the model that performs the best for this particular prediction task can be identified.

All features underwent scaling using the StandardScaler(). This method ensured features were on a similar scale, centering them around zero with a standard deviation of one. To address dataset size limitation and potential class imbalance, ADASYN (Adaptive Synthetic Sampling) was chosen as our oversampling technique. This method was preferred over others like RandomOverSampler due to its ability to generate synthetic samples in regions where the data distribution is sparse. This adaptive approach minimized the risk of overfitting while effectively balancing the class distribution.

To improve the predictive efficacy of the model, five time nodes were set with a spacing of 1 in the range of 1–5 years. For patients who experience recurrence within the first year, we will still incorporate them into the model development in the second year. This was because our time nodes is measured in “k” years, rather than specifically in the “kth” year. This decision was based on the clinical significance of predicting a patient's recurrence in a few years, and providing an intuitive and dynamic recurrence curve, rather than solely predicting recurrence in a specific year. From our original dataset, two key variables were present: “recurrence” (a binary indicator) and “recurrence_time” (quantified in months). Utilizing these, we generated our target variables, “recurrence_in_k_years”.

All 84 features were retained in the model to ensure comprehensive data capture and to avoid the premature exclusion of potentially relevant predictors. The reliance on advanced algorithms such as XGB and LightGBM, known for their proficiency in handling high-dimensional data, further justified this decision. The study of feature importance was not conducted for optimization purposes, but rather to provide clinically relevant insights. By understanding which features were deemed most influential by the models, valuable information can be provided to the clinical community about the factors crucial for predicting disease recurrence. Recognizing the distinct consequences of false negatives vs. false positives in medical scenarios, we additionally assigned a cost ratio for False Positives (FP) to False Negatives (FN) of 1:4. This emphasizes the criticality of not overlooking potential risks, as missing a true positive case can have significant ramifications. Beyond the cost matrix, all models were utilized with default configuration.

#### 2.5.3 K-fold cross validation

Concerning our methodology of using only a training and a testing set, without a dedicated validation set, we had specific considerations. Given the limited size of our dataset, we believed that allocating a portion to a validation set could adversely impact the model's performance. Moreover, research indicated that with small datasets, the models often perform best with default hyperparameters, and that hyperparameter tuning might negatively influence performance (14, 15). These factors led us to the decision of not engaging in hyperparameter tuning and adpoting a k-fold Cross Validation with *k* = 10. Our testing set, being independent from the training set, serves to effectively evaluate the model's performance on unseen data.

In cross validation, training set was split randomly into 10 folds. For each iteration, 9 of the 10 folds were used as training set and 1 as validation set. An average AUC was calculated for each model to evaluate if the model was overfitted and used as a benchmark for the model's performance on the testing set. XGBoost and LightGBM consistently outperformed other models in every time node, with AUC of 83.97% and 83.02%, indicating a solid performance of our model and no sign of overfitting. Since the difference between XGBoost and LightGBM is trivial, we decided to conduct final model selection based on their performance on testing set.

#### 2.5.4 Performance evaluation

For each time node, the performance of each model was compared, and the comprehensive evaluation indices were AUC, sensitivity, specificity, accuracy, and F2 score. Considering the ability of the AUC score to evaluate the performance of a model across all thresholds, it was used as a single metric to select the best model at each time node and the model with the highest performance. These metrics were also compared with those of k-fold Cross Validation, to see if the model was overfitted to the training set, in which condition, metrics of validation would be significantly higher than those of testing set.

Descriptive statistics and machine learning analyses were performed using SPSS version 23.0 (IBM Corp, Armonk, NY, USA) and Python version 3.6.15 (Python Software Foundation, Wilmington, DE, USA).

## 3 Results

### 3.1 Patient basic characteristics and clinical outcomes

**Figure 1:**
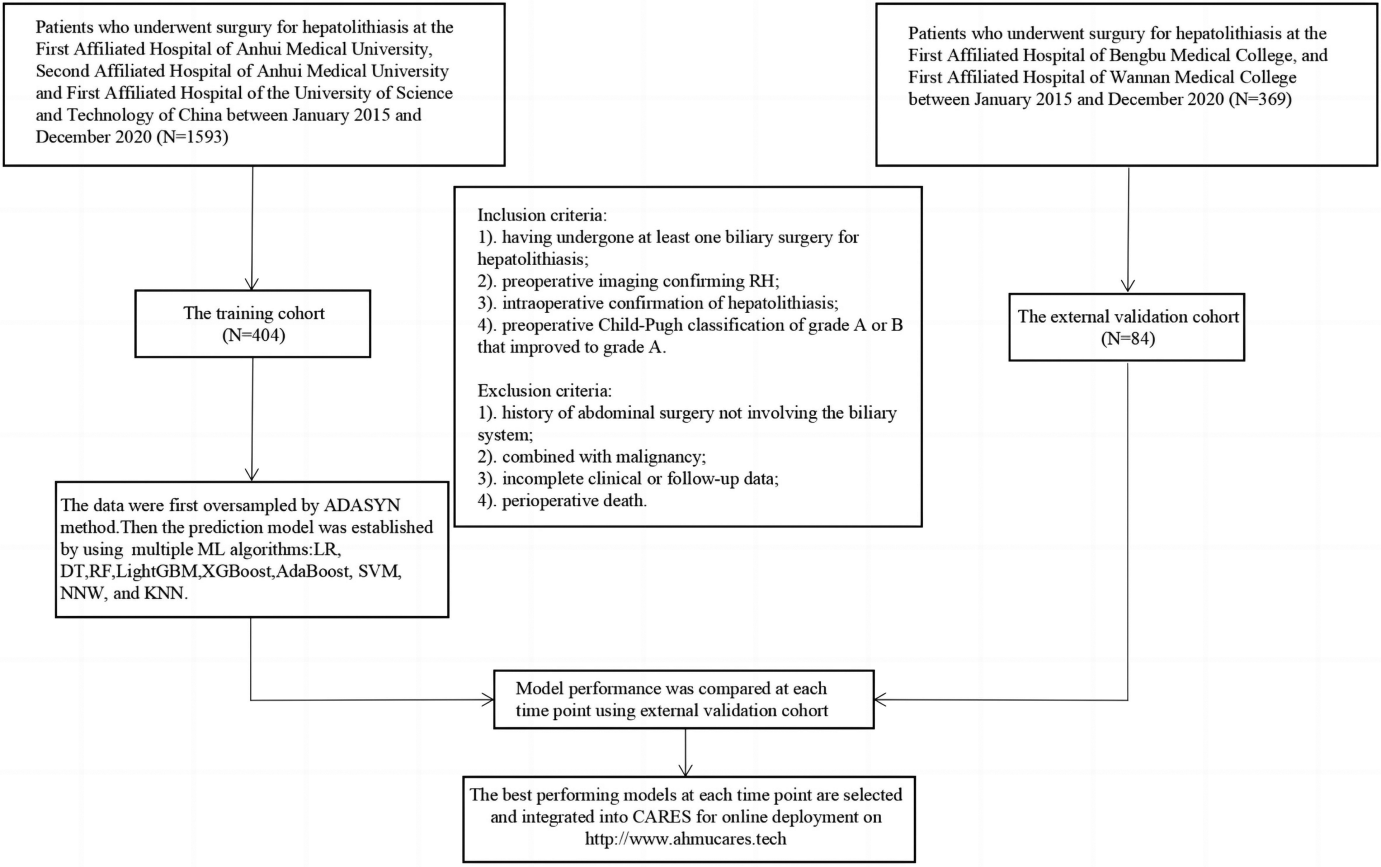
Flow chart of patient enrollment. RH, recurrent hepatolithiasis; ML, machine learning; XGBoost, extreme gradient boosting; LightGBM, light gradient-boosting machine; RF, random forest; SVM, support vector machine; AdaBoost, adaptive boosting; NNW, neural network; DT, decision tree; LR, logistic regression; KNN, K-nearest neighbour; CARES, correlation Analysis and Recurrence Evaluation System.

Based on these criteria, the data of 488 patients who underwent hepatolithiasis surgery in the five medical centres during the 5-year period were evaluated, with 294 patients admitted at the First Affiliated Hospital of Anhui Medical University, 51 patients admitted at the Second Affiliated Hospital of Anhui Medical University, 59 patients admitted at the First Affiliated Hospital of the University of Science and Technology of China, 32 patients admitted at the First Affiliated Hospital of Bengbu Medical College, and 52 patients admitted at the First Affiliated Hospital of Wannan Medical College (Figure 1).

Overall, 488 patients were included in the ML model [mean age, 57.9 ± 12.0 years; >60 years, *n* = 235 (48.2%); female, *n* = 331, 67.8%]. A total of 157 patients (32.1%) underwent more than one surgical treatment, and 89 patients (18.2%) underwent hepatectomy. The characteristics of the training and testing sets were not significantly different (Table 1). A total of 135 patients (27.7%) had a recurrence within 5 years (Table 2). All predictor variables were incorporated into the ML model to predict the risk of recurrence in patients with RH.

**Table 1:** Preoperative clinical characteristics of patients with recurrent hepatolithiasis after surgery.

Characteristic	Total (*n* = 488)	Training set (*n* = 404)	Testing set (*n* = 84)	Statistic	*P* value
Gender, *n* (%)				*χ*^2^ = 0.257	0.612
Male	157 (32.17)	128 (31.68)	29 (34.52)		
Female	331 (67.83)	276 (68.32)	55 (65.48)		
Age, *n* (%)				*χ*^2^ = 1.520	0.218
<60	239 (48.98)	203 (50.25)	36 (42.86)		
≥60	249 (51.02)	201 (49.75)	48 (57.14)		
BMI, mean ± SD	21.97 ± 2.85	21.93 ± 2.89	22.17 ± 2.67	*t* = −0.700	0.484
Abdominal pain, *n* (%)				*χ*^2^ = 0.000	0.987
No	70 (14.34)	58 (14.36)	12 (14.29)		
Yes	418 (85.66)	346 (85.64)	72 (85.71)		
Fever, *n* (%)				*χ*^2^ = 0.284	0.594
No	303 (62.09)	253 (62.62)	50 (59.52)		
Yes	185 (37.91)	151 (37.38)	34 (40.48)		
Emesis, *n* (%)				*χ*^2^ = 2.067	0.151
No	383 (78.48)	322 (79.70)	61 (72.62)		
Yes	105 (21.52)	82 (20.30)	23 (27.38)		
Icterus, *n* (%)				*χ*^2^ = 0.722	0.395
No	384 (78.69)	315 (77.97)	69 (82.14)		
Yes	104 (21.31)	89 (22.03)	15 (17.86)		
Pressing pain, *n* (%)				*χ*^2^ = 0.776	0.378
No	311 (63.73)	261 (64.60)	50 (59.52)		
Yes	177 (36.27)	143 (35.40)	34 (40.48)		
Smoking, *n* (%)				*χ*^2^ = 0.449	0.503
No	400 (81.97)	329 (81.44)	71 (84.52)		
Yes	88 (18.03)	75 (18.56)	13 (15.48)		
Drinking, *n* (%)				*χ*^2^ = 0.491	0.483
No	418 (85.66)	344 (85.15)	74 (88.10)		
Yes	70 (14.34)	60 (14.85)	10 (11.90)		
Number_of_operations, *n* (%)				Fisher	0.399
1	331 (67.83)	278 (68.81)	53 (63.10)		
2	100 (20.49)	77 (19.06)	23 (27.38)		
3	47 (9.63)	40 (9.90)	7 (8.33)		
≥4	10 (2.05)	9 (2.23)	1 (1.19)		
Previous hepatectomy, *n* (%)				*χ*^2^ = 2.159	0.142
No	175 (35.86)	139 (34.41)	36 (42.86)		
Yes	313 (64.14)	265 (65.59)	48 (57.14)		
Liver cirrhosis, *n* (%)				*χ*^2^ = 3.785	0.052
No	428 (87.7)	349 (86.39)	79 (94.05)		
Yes	60 (12.3)	55 (13.61)	5 (5.95)		
Surgical method, *n* (%)				*χ*^2^ = 2.477	0.116
Open surgery	436 (89.34)	365 (90.35)	71 (84.52)		
Laparoscopic surgery	52 (10.66)	39 (9.65)	13 (15.48)		
Intrahepatic narrow, *n* (%)				*χ*^2^ = 1.130	0.288
No	367 (75.2)	300 (74.26)	67 (79.76)		
Yes	121 (24.8)	104 (25.74)	17 (20.24)		
Hepatic lobe atrophy, *n* (%)				*χ*^2^ = 0.231	0.630
No	215 (44.06)	176 (43.56)	39 (46.43)		
Yes	273 (55.94)	228 (56.44)	45 (53.57)		
AGR, *n* (%)				*χ*^2^ = 0.671	0.413
>1.5	158 (32.38)	134 (33.17)	24 (28.57)		
≤1.5	330 (67.62)	270 (66.83)	60 (71.43)		
NLR, *n* (%)				*χ*^2^ = 3.156	0.076
<2.462	292 (59.84)	249 (61.63)	43 (51.19)		
≥2.462	196 (40.16)	155 (38.37)	41 (48.81)		
PLR, *n* (%)				*χ*^2^ = 0.168	0.682
<173.74	393 (80.53)	324 (80.20)	69 (82.14)		
≥173.74	95 (19.47)	80 (19.80)	15 (17.86)		
TBIL, *n* (%)				*χ*^2^ = 0.071	0.790
<34.2	400 (81.97)	332 (82.18)	68 (80.95)		
≥34.2	88 (18.03)	72 (17.82)	16 (19.05)		
ALT, *n* (%)				*χ*^2^ = 0.728	0.393
<50	299 (61.27)	251 (62.13)	48 (57.14)		
≥50	189 (38.73)	153 (37.87)	36 (42.86)		
AST, *n* (%)				*χ*^2^ = 0.008	0.929
<40	300 (61.48)	248 (61.39)	52 (61.90)		
≥40	188 (38.52)	156 (38.61)	32 (38.10)		
ALP, *n* (%)				*χ*^2^ = 0.899	0.343
<200	309 (63.32)	252 (62.38)	57 (67.86)		
≥200	179 (36.68)	152 (37.62)	27 (32.14)		
GGT, *n* (%)				*χ*^2^ = 0.192	0.661
<150	243 (49.8)	203 (50.25)	40 (47.62)		
≥150	245 (50.2)	201 (49.75)	44 (52.38)		
CA19-9, *n* (%)				*χ*^2^ = 2.288	0.130
<34	338 (69.26)	274 (67.82)	64 (76.19)		
≥34	150 (30.74)	130 (32.18)	20 (23.81)		

This table summarizes patient data on key clinically significant variables only. BMI, body mass index; AGR, albumin-to-globulin ratio; NLR, neutrophil-to-lymphocyte ratio; PLR, platelet-to-lymphocyte ratio; TBIL, total bilirubin; ALT, alanine aminotransferase; AST, aspartate aminotransferase; ALP, alkaline phosphatase; GGT, γ-glutamyl transpeptidase; CA19-9, carbohydrate antigen19-9;.

**Table 2:** The number of recurrent patients in k years.

In k years	1	2	3	4	5
Number	44	108	126	132	135

In Table 1, we have presented the preoperative clinical characteristics of the patients in a simplified categorical or hierarchical manner for clarity and ease of understanding for the readers. Please note that during the actual model-building process, the original continuous values of these variables were utilized. We believe using the continuous data during model-building aids in capturing subtle nuances and providing a more accurate representation, whereas the categorized data in the table helps in presenting an easier-to-read overview.

### 3.2 Model performance

**Figure 2:**
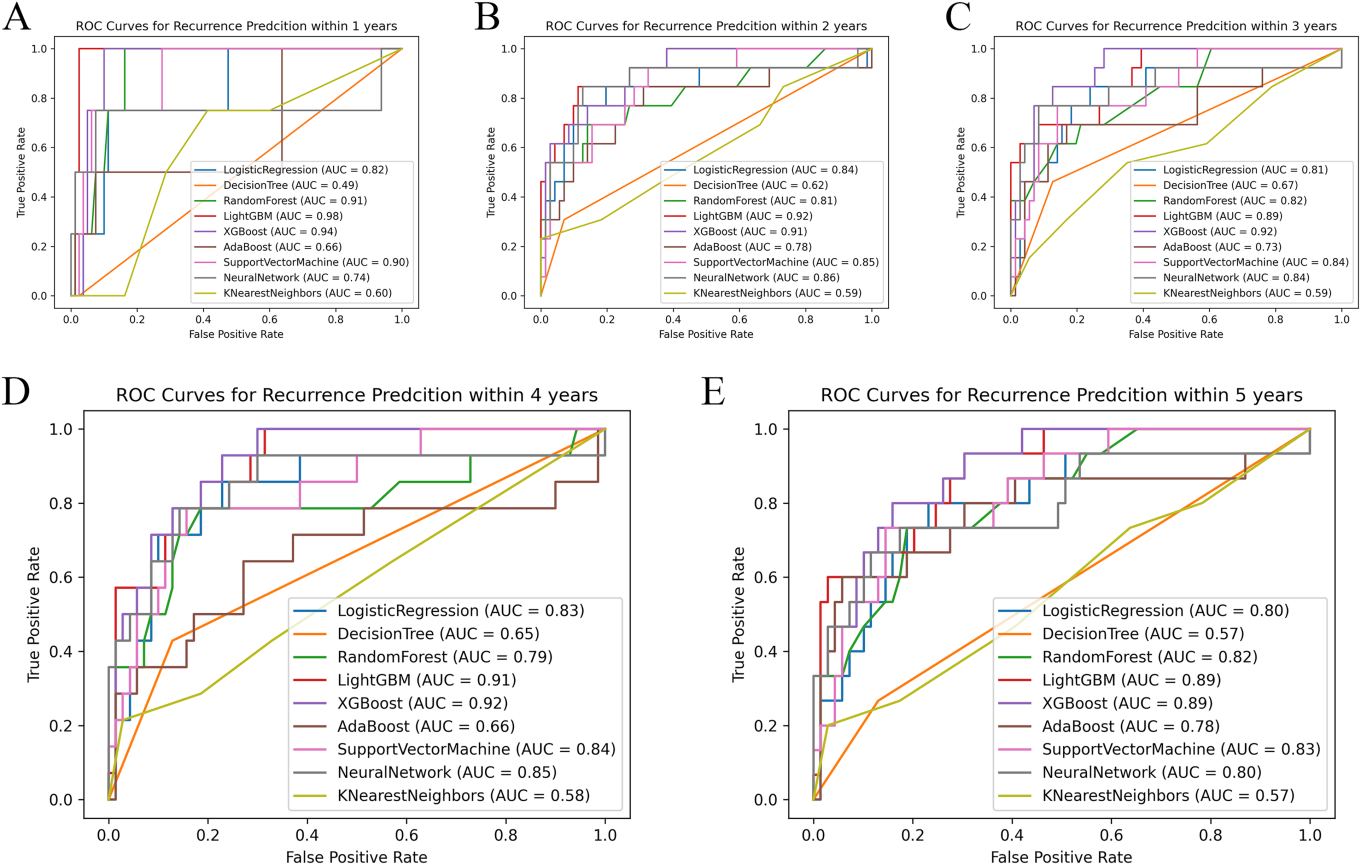
Comparison of ROC curves of each model at different time nodes. Panels **A–E** respectively show the ROC curves and AUC of each model at the time points set to 1, 2, 3, 4, and 5 years. AUC, area under the receiver operating characteristic curve.

The nine models were built and externally validated. The AUC values of the models are presented in Table 3. In terms of predicting RH recurrence at 3 years and more, XGBoost showed optimal performance, with AUCs of about 0.9 or greater, which fully demonstrates its strength. It can efficiently and flexibly handle multivariate data and assemble weak prediction models to build an accurate one (16, 17). In the prediction of recurrence within 1 year and 2 years, LightGBM was more advantageous, with AUCs of 0.981 and 0.924, respectively, whereas the performance of the DT and KNN models was unsatisfactory, probably because the sample size was not sufficiently large (Figure 2) (18). It was worth noticing that model showed better performance on testing set than validation, indicating that it was not overfitted to the training set.

**Table 3:** Area under the receiver operating characteristic curve (AUC) of each model at different time nodes.

Model	AUC within 1 year	AUC within 2 years	AUC within 3 years	AUC within 4 years	AUC within 5 years
XGBoost	0.941	0.906	0.922	0.917	0.887
LightGBM	0.981	0.924	0.889	0.907	0.885
RF	0.903	0.825	0.852	0.849	0.774
SVM	0.900	0.856	0.836	0.843	0.832
AdaBoost	0.659	0.779	0.732	0.661	0.781
NNW	0.747	0.852	0.823	0.845	0.813
DT	0.469	0.650	0.674	0.636	0.542
LR	0.819	0.839	0.810	0.833	0.795
KNN	0.600	0.592	0.585	0.576	0.568

This table summarizes area under the receiver operating characteristic curve (AUC) of each model at different time nodes only. Additional data on optimal parameters and performance of each model is summarized in Supplementary Appendix. XGBoost, extreme gradient boosting; LightGBM, light gradient-boosting machine; RF, random forest; SVM, support vector machine; AdaBoost, adaptive boosting; NNW, neural network; DT, decision tree; LR, logistic regression; KNN, K-nearest neighbour.

**Figure 3:**
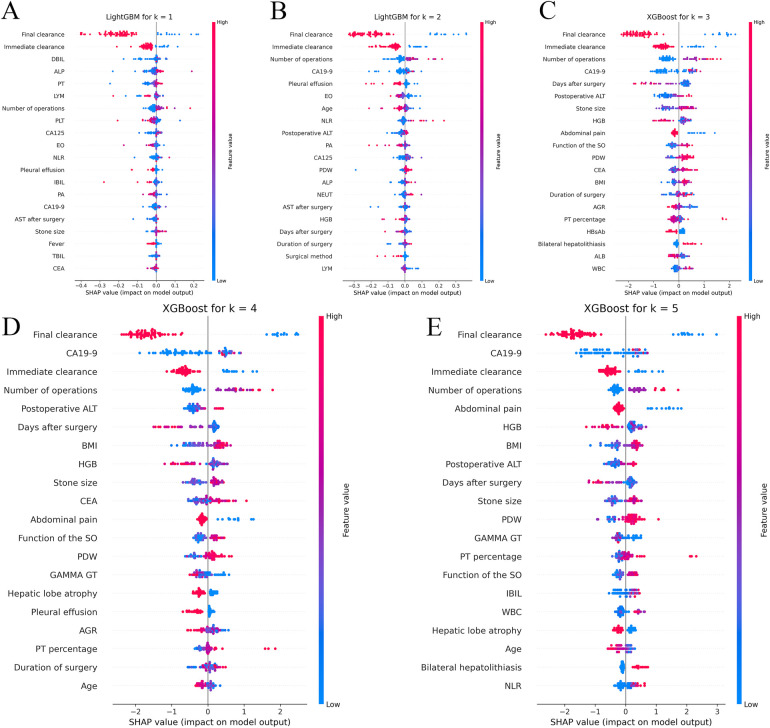
Shapley additive explanations (SHAP) analyses of the best-performing machine learning models for predicting recurrence of hepatolithiasis. Panels **A,B** respectively show the Shapley additive explanations (SHAP) for the LightGBM model, which performed the best at the 1-year and 2-year time points, while panels **C–E** respectively show the SHAP for the XGBoost model at the 3-year, 4-year, and 5-year time points. XGBoost, extreme gradient boosting; LightGBM, light gradient-boosting machine; DBIL, direct bilirubin; ALP, alkaline phosphatase; PT, prothrombin time; LYM, lymphocyte; PLT, platelet count; CA125, carbohydrate antigen 125; EO, eosinophil; NLR, neutrophil-to-lymphocyte ratio; IBIL, indirect bilirubin; PA, prealbumin; CA19-9, carbohydrate antigen19-9; AST, aspartate aminotransferase; TBIL, total bilirubin; CEA, carcinoembryonic antigen; PDW, platelet distribution width; NEUT, neutrophil count; AGR, albumin-to-globulin ratio; HBsAb, hepatitis B surface antibody; WBC, white blood cell; BMI, body mass index; HGB, hemoglobin; GGT, γ-glutamyl transpeptidase.

For the clinical results at each time point, Shapley Additive Explanations (SHAP) were generated to construct a comprehensive explainable framework showing the importance and direction of each predictor variable, increasing the interpretability of the model. The position of each predictor variable on the *y*-axis was ranked in order of relative importance, with the most important predictor variable at the top. For each predictor variable, the position of each point on the *x*-axis (red indicates higher values or the presence of binary factors) represents the contribution of the individual participant to the overall SHAP value, with highly positive contributions on the far right (Figure 3).

### 3.3 Predictive analysis and clinical application

Instant and final clearance were of considerable importance in the prediction of almost every time point, whereas the number of previous surgeries and the neutrophil/lymphocyte ratio were also of great importance, which is in line with our previous findings (11). Moreover, advanced ML models can capture higher-order non-linear interactions among predictors; therefore, we also found many previously unappreciated or undetected factors that have great impact on recurrence, such as the function of the sphincter of Oddi (SO), carbohydrate antigen 19-9 (CA19-9), symptom score, and platelet count.

The system named CARES employs five specialized models, each optimized for predicting the risk of disease recurrence for years 1–5 post-surgery. Specifically, CARES has 5 system components and goes through the following steps.

Firstly, for each k (ranging from 1 to 5), a dedicated model is trained using the entire dataset to predict the probability of a patient experiencing disease recurrence k years after surgery. This results in 5 distinct models, each optimized for its specific prediction year. Secondly, for a new patient, measurements and relevant clinical information serve as the input. In instances where certain data points are missing, these are substituted with the sample average to ensure a comprehensive data input. Thirdly, each of the 5 models processes the input data, providing individual probability estimates of the patient's risk of disease recurrence for years 1 through 5. Fourthly, to ensure that the risk curve exhibits clinical coherence (i.e., the risk doesn't drop in subsequent years, which would be counterintuitive), an isotonic regression is applied to the predicted probabilities. Lastly, the output of the CARES system is a graphical representation or “risk curve”. This curve offers a clear visualization of a patient's estimated risk of recurrence across the 5-year period post-surgery.

**Figure 4:**
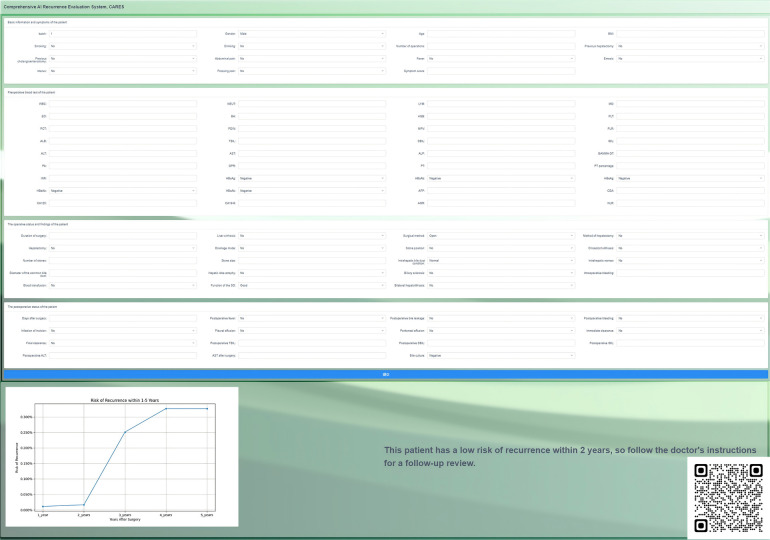
Page presentation of the online correlation analysis and recurrence evaluation system (CARES), which is available for free at http://www.ahmucares.tech:5000/ or by scanning the QR code.

This system was encapsulated and deployed online. When the user inputs the patient's predictors, it outputs a curve of recurrence risk over time; when the patient's recurrence risk is higher at a certain time point or spikes at a certain period of time, we notify the user of the output on the output graph to draw attention to the patient's recurrence risk (Figure 4). This incorporation of individual and aggregated predictive models aids in offering a comprehensive and nuanced risk profile. Compared with previous scoring systems, our calculator is easier to use and the output is more intuitive, with greater utility and a higher predictive value. The CARES is available for free online (19) and can also be accessed by scanning the QR code.

In terms of evaluation, the model's efficacy can be gauged by comparing its predictions against actual recurrence events in a real-world clinical setting. After deployment in real practice, continual validation and recalibration can further refine the model, ensuring its sustained relevance and accuracy.

## 4 Discussion

### 4.1 Principal findings

In this study, ML methods and multicentre clinical data were combined to build CARES, an accurate, efficient, and user-friendly prediction model that integrates clinical characteristics to predict the dynamic recurrence risk of RH after surgery, and then analysed the risk factors that may be associated with recurrence using the SHAP method. Based on SHAP at various time points, immediate stone clearance, final stone clearance, number of previous surgeries, and preoperative CA19-9 index were the most significant predictors of recurrence after reoperation in RH patients. We employed state-of-the-art algorithms, such as XGB and LightGBM. It's noteworthy that, to our knowledge, these algorithms have not been previously utilized in modeling recurrence of this specific disease. CARES is the first model that uses ML to assess the prognosis of patients with RH after biliary surgery. We incorporated the latest dataset available, which, to the best of our knowledge, is unparalleled in its scale and comprehensiveness for this subject.

### 4.2 Interdisciplinary integration

Hepatolithiasis is a relatively common benign disease in East Asia; however, the management of patients with hepatolithiasis has been challenging owing to the high rates of treatment failure, recurrence, and complications (20–22). Patients with RH are also more difficult to re-treat because they have already undergone one or multiple surgeries, and repeat surgery places a greater psychological and financial burden on patients. Therefore, a model that accurately predicts the individual dynamic recurrence risk of patients with RH after surgical treatment could provide great value in guiding the assessment of postoperative efficacy as well as the development of a follow-up strategy (23).

The application of AI in healthcare is growing rapidly with potential applications in various subspecialties and subfields (24–26). As an important branch of AI, ML can be trained by inputting large amounts of labelled data (27) and analysing these data to identify relevant patterns that can then be used to predict future events or states (28). It has the ability to learn automatically from data and algorithms and uses past experience to improve performance (29). Unlike traditional regression-based methods, ML algorithms capture higher-order non-linear interactions between predictors (30) and thus focus on detecting hard-to-recognise patterns in complex data. CARES allows the comparison of multiple learning algorithms to identify the algorithm with the best performance.

When developing CARES, a different oversampling method was used, ADASYN, to prevent the imbalance in the amount of negative vs. positive data from distorting the model's performance. Unlike random oversampling, which simply replicates existing examples, ADASYN generates new synthetic examples in a small number of classes that are slightly different from existing examples, with a particular focus on samples that are more difficult to learn. These synthetic examples make the model more robust and reduce the risk of overfitting because they introduce more variability and help the model to better generalise the training data to new data.

Our study also demonstrated that a prediction model based on ML techniques was superior to the traditional regression analysis method in terms of predictive performance. Previous studies had few predictive models for postoperative recurrence in patients with RH. We used traditional LR to build a recurrence prediction model for patients with RH after biliary surgery, which had an AUC of 0.754 and was not fully satisfactory (11). In contrast, with the help of ML techniques, the AUC of LightGBM reached 0.981 and 0.924 for patients with recurrence within 1 year and 2 years after surgery, respectively, whereas XGBoost performed exceptionally well for patients with recurrence at 3 years and beyond, with AUCs of 0.922, 0.917, and 0.887 at 3, 4, and 5 years, respectively.

As a widely used model in biological and medical analyses, XGBoost is a boosting algorithm with many advantages. First, several variables may have affected disease recurrence. By building an ensemble of decision trees, XGBoost can capture complex relationships between features and outcomes, which may be particularly important in medical scenarios where multiple factors interact to influence outcomes. Second, our dataset contains a large number of predictor variables, including binary, numerical, and categorical data. XGBoost can handle all these types of data, allowing us to incorporate all potentially relevant information into the prediction (31). Finally, our dataset was considered unbalanced, with a limited number of samples and fewer positive data. XGBoost addresses this issue. It also provides resilience against overfitting and supports parallel processing to maximise the use of resources (32). Therefore, XGBoost tends to have excellent performance when the number of predictor variables is large and the dataset is not balanced. The present study also indicated that the prediction model based on XGBoost had the best performance.

As ML becomes more computationally powerful and the complexity of models increases, understanding the underlying logic and decision factors of the models becomes increasingly difficult. Therefore, enhancing the interpretability of black boxes so that people can understand the reasons for their predictions can considerably improve the applicability and credibility of models (33). Therefore, we combined the predictions of CARES with SHAP to construct a comprehensive explanatory framework for presenting the contribution of each predictor variable to the results and to increase the transparency of the model (34). SHAP has many advantages. It can calculate the contribution of various factors, determine the positivity or negativity of each contribution, quantify each factor's contribution to the stone recurrence/non-occurrence probability, and predict recurrence without decreasing the predictive model's accuracy (33, 35). These advantages are important for the prediction of potential recurrence risk, clinical focus of influencing factors, and interpretation of CARES prediction results.

### 4.3 Clinical findings and contributions

According to the results of the SHAP, instant and final clearance of stones were the most important predicting factors. Patients who fail to achieve instant clearance and final clearance appear to be at a much higher risk of recurrence, showing that perfect preoperative examination and fine intraoperative operation are quite beneficial in improving the patient's prognosis. Therefore, the surgical method should be carefully selected to remove all stones intraoperatively, based on preoperative examination. For patients in whom intraoperative stone extraction is difficult, such as those with stones in both the hepatic and biliary ducts, severe lateral hepatectomy combined with choledochoscopic lithotripsy can be attempted to obtain a high stone removal rate (36, 37). Stones that are difficult to remove intraoperatively should be removed postoperatively using trans-T-tube sinusoidal choledochoscopy.

The number of previous surgeries was also a major concern. According to the SHAP, a greater number of previous surgeries significantly increases a patient's risk of recurrence. According to previous studies, up to 95% of prior abdominal surgeries result in intra-abdominal adhesions (38), which may be related to intraoperative vascular and intestinal injuries (39). A complex abdominal environment can greatly increase the difficulty of surgery, making accurate resection of lesions and removal of stones difficult. Therefore, care should be taken when choosing a surgical procedure for patients who have undergone multiple laparotomies. Open approach may be a better option than laparoscopic approach because in patients with severe abdominal adhesions, improper placement of the trocar may prevent effective laparoscopic surgery and may damage the viscera or vascular around the adhesions. Loosening the abdominal adhesions to accurately identify the anatomical landmarks can be a challenge during surgery.

In our study, CA19-9 played an important role in recurrence at certain time points, higher CA19-9 levels in patients on preoperative examination suggested a higher risk of recurrence. Previous studies on the relationship between CA19-9 and hepatolithiasis have often been limited to whether it is associated with malignancy in biliary diseases; little research has been conducted on its relationship with recurrence. According to Ker *et al*. (40), the concentration of CA19-9 is not only affected by tumours but is also increased by severe infections in patients with hepatolithiasis. Cases of stone-induced acute bile duct inflammation leading to elevated CA19-9 levels were also reported by Sheen-Chen *et al*. (41). We hypothesised that patients with elevated CA19-9 levels may have more severe tract infections, which may disrupt the biliary environment and increase the risk of recurrence.

In addition to the aforementioned key risk factors, the function of SO also affected recurrence in our prediction model. The primary function of the SO is to regulate bile influx into the duodenum and to prevent duodenal reflux (42). Duodenal reflux of food debris can lead to Escherichia coli infections and a decrease in biliary pH. E. coli can generate β-glucuronidase, which hydrolyses water-soluble direct bilirubin into water-insoluble indirect bilirubin, thereby facilitating stone formation in the biliary tract (43). Consequently, patients with poorer SO function are more prone to recurrence. Therefore, maintaining the functional integrity of SO helps to reduce the recurrence rate in patients with RH. In patients with normal SO function, the best method of biliary drainage is T-tube drainage, which is relatively simple, has a high stone-clearance rate, and preserves the structural integrity and continuity of the extrahepatic bile ducts because it preserves SO function. T-tube drainage significantly reduces the incidence of post-operative reflux cholangitis in patients with normal SO function. However, in patients with complete loss of function or stenosis of the SO, Roux-en-Y hepatico-jejunostomy is one of the best methods available for biliary drainage. Roux-en-Y hepatico-jejunostomy has the advantage that it reduces reflux of duodenal fluid, but this procedure abandons the SO (44). Therefore, to reduce the recurrence rate in patients with RH, the surgeon should carefully choose the method for different states of SO function and preserve SO function as much as possible to prevent the occurrence of reflux cholangitis.

Naturally, other factors seem to influence the recurrence of hepatolithiasis, but the direct link between these factors, such as postoperative fever, and the recurrence of hepatolithiasis is difficult to understand. However, ML has the advantage of observing complex, multidimensional, and non-linear relationships between different predictor variables in biological systems. Perhaps in the future, we can aim to understand how these factors cause physiological and pathological “butterfly effects” in the human body and isolate them to demonstrate a complete “chain of evidence.”

To improve the application value of the model, we encapsulated the CARES as a recurrence risk curve calculator and deployed it online. By inputting patient information, the calculator outputs a dynamic recurrence risk curve that increases with time after the operation, and the user can approximate the patient's possible risk of recurrence based on the output. An open interface is reserved in CARES for interfacing with the hospital information system.

CARES not only has a better performance but can also visually output the change in recurrence risk of patients in each period from 1 to 5 years after surgery, suggesting the period when doctors and patients need to be extra cautious, as well as the indicators and guidelines that they need to focus on.

### 4.4 Limitations

This study has some limitations. First, the retrospective nature of the methodology may lead to a selection bias, and prospective studies are needed to validate the accuracy of the results. Second, during model training, due to the imbalanced nature of our dataset, we adapted ADASYN as oversampler. We acknowledged that while ADASYN helped address class imbalance, it may not fully capture the complexities of real-world distributions in clinical settings. Third, the explainable internal working logic of the model remains one of the biggest barriers to implementing cutting-edge ML techniques in biomedical research. We must better understand the evolving and complex relationships between physicians and smart tools in clinical settings to provide better treatment strategies for patients.

## 5 Conclusions

Multiple ML algorithms were used to construct CARES, which integrates various clinical data to predict the dynamic recurrence risk of RH patients after surgery. The predictive power of our model was externally validated based on a multicentre database. We believe that CARES can provide critical prognostic predictions for patients after RH surgery and may facilitate more efficient clinical decision-making by surgeons and patients.

## Data Availability

The raw data supporting the conclusions of this article will be made available by the authors, without undue reservation.
