# Supplementary material for: Correlation analysis and recurrence evaluation system for patients with recurrent hepatolithiasis: a multicentre retrospective study
Source: Front Digit Health. 2024 Nov 27;6:1510674. doi: 10.3389/fdgth.2024.1510674 (PMC11631919; doi:10.3389/fdgth.2024.1510674)
Supplement: Supplementary file 1 [file Table1.docx]

**Table 1.** Preoperative clinical characteristics of patients with recurrent hepatolithiasis after surgery

| **Characteristic** | **Total (n = 488)** | **training set**  **(n = 404)** | **testing set**  **(n = 84)** | **statistic** | **P value** |
| --- | --- | --- | --- | --- | --- |
| Gender, n (%) |  |  |  | χ²=0.257 | 0.612 |
| Male | 157 (32.17) | 128 (31.68) | 29 (34.52) |  |  |
| Female | 331 (67.83) | 276 (68.32) | 55 (65.48) |  |  |
| Age, n (%) |  |  |  | χ²=1.520 | 0.218 |
| <60 | 239 (48.98) | 203 (50.25) | 36 (42.86) |  |  |
| ≥60 | 249 (51.02) | 201 (49.75) | 48 (57.14) |  |  |
| BMI, Mean ± SD | 21.97 ± 2.85 | 21.93 ± 2.89 | 22.17 ± 2.67 | t=-0.700 | 0.484 |
| Abdominal Pain, n (%) |  |  |  | χ²=0.000 | 0.987 |
| No | 70 (14.34) | 58 (14.36) | 12 (14.29) |  |  |
| Yes | 418 (85.66) | 346 (85.64) | 72 (85.71) |  |  |
| Fever, n (%) |  |  |  | χ²=0.284 | 0.594 |
| No | 303 (62.09) | 253 (62.62) | 50 (59.52) |  |  |
| Yes | 185 (37.91) | 151 (37.38) | 34 (40.48) |  |  |
| Emesis, n (%) |  |  |  | χ²=2.067 | 0.151 |
| No | 383 (78.48) | 322 (79.70) | 61 (72.62) |  |  |
| Yes | 105 (21.52) | 82 (20.30) | 23 (27.38) |  |  |
| Icterus, n (%) |  |  |  | χ²=0.722 | 0.395 |
| No | 384 (78.69) | 315 (77.97) | 69 (82.14) |  |  |
| Yes | 104 (21.31) | 89 (22.03) | 15 (17.86) |  |  |
| Pressing pain, n (%) |  |  |  | χ²=0.776 | 0.378 |
| No | 311 (63.73) | 261 (64.60) | 50 (59.52) |  |  |
| Yes | 177 (36.27) | 143 (35.40) | 34 (40.48) |  |  |
| Smoking, n (%) |  |  |  | χ²=0.449 | 0.503 |
| No | 400 (81.97) | 329 (81.44) | 71 (84.52) |  |  |
| Yes | 88 (18.03) | 75 (18.56) | 13 (15.48) |  |  |
| Drinking, n (%) |  |  |  | χ²=0.491 | 0.483 |
| No | 418 (85.66) | 344 (85.15) | 74 (88.10) |  |  |
| Yes | 70 (14.34) | 60 (14.85) | 10 (11.90) |  |  |
| Number_of_operations, n (%) |  |  |  | Fisher | 0.399 |
| 1 | 331 (67.83) | 278 (68.81) | 53 (63.10) |  |  |
| 2 | 100 (20.49) | 77 (19.06) | 23 (27.38) |  |  |
| 3 | 47 (9.63) | 40 (9.90) | 7 (8.33) |  |  |
| ≥4 | 10 (2.05) | 9 (2.23) | 1 (1.19) |  |  |
| Previous hepatectomy, n (%) |  |  |  | χ²=2.159 | 0.142 |
| No | 175 (35.86) | 139 (34.41) | 36 (42.86) |  |  |
| Yes | 313 (64.14) | 265 (65.59) | 48 (57.14) |  |  |
| Liver cirrhosis, n (%) |  |  |  | χ²=3.785 | 0.052 |
| No | 428 (87.7) | 349 (86.39) | 79 (94.05) |  |  |
| Yes | 60 (12.3) | 55 (13.61) | 5 (5.95) |  |  |
| Surgical method, n (%) |  |  |  | χ²=2.477 | 0.116 |
| Open surgery | 436 (89.34) | 365 (90.35) | 71 (84.52) |  |  |
| Laparoscopic surgery | 52 (10.66) | 39 (9.65) | 13 (15.48) |  |  |
| Intrahepatic narrow, n (%) |  |  |  | χ²=1.130 | 0.288 |
| No | 367 (75.2) | 300 (74.26) | 67 (79.76) |  |  |
| Yes | 121 (24.8) | 104 (25.74) | 17 (20.24) |  |  |
| Hepatic lobe atrophy, n (%) |  |  |  | χ²=0.231 | 0.630 |
| No | 215 (44.06) | 176 (43.56) | 39 (46.43) |  |  |
| Yes | 273 (55.94) | 228 (56.44) | 45 (53.57) |  |  |
| AGR, n (%) |  |  |  | χ²=0.671 | 0.413 |
| ＞1.5 | 158 (32.38) | 134 (33.17) | 24 (28.57) |  |  |
| ≤1.5 | 330 (67.62) | 270 (66.83) | 60 (71.43) |  |  |
| NLR, n (%) |  |  |  | χ²=3.156 | 0.076 |
| <2.462 | 292 (59.84) | 249 (61.63) | 43 (51.19) |  |  |
| ≥2.462 | 196 (40.16) | 155 (38.37) | 41 (48.81) |  |  |
| PLR, n (%) |  |  |  | χ²=0.168 | 0.682 |
| <173.74 | 393 (80.53) | 324 (80.20) | 69 (82.14) |  |  |
| ≥173.74 | 95 (19.47) | 80 (19.80) | 15 (17.86) |  |  |
| TBIL, n (%) |  |  |  | χ²=0.071 | 0.790 |
| <34.2 | 400 (81.97) | 332 (82.18) | 68 (80.95) |  |  |
| ≥34.2 | 88 (18.03) | 72 (17.82) | 16 (19.05) |  |  |
| ALT, n (%) |  |  |  | χ²=0.728 | 0.393 |
| <50 | 299 (61.27) | 251 (62.13) | 48 (57.14) |  |  |
| ≥50 | 189 (38.73) | 153 (37.87) | 36 (42.86) |  |  |
| AST, n (%) |  |  |  | χ²=0.008 | 0.929 |
| <40 | 300 (61.48) | 248 (61.39) | 52 (61.90) |  |  |
| ≥40 | 188 (38.52) | 156 (38.61) | 32 (38.10) |  |  |
| ALP, n (%) |  |  |  | χ²=0.899 | 0.343 |
| <200 | 309 (63.32) | 252 (62.38) | 57 (67.86) |  |  |
| ≥200 | 179 (36.68) | 152 (37.62) | 27 (32.14) |  |  |
| GGT, n (%) |  |  |  | χ²=0.192 | 0.661 |
| <150 | 243 (49.8) | 203 (50.25) | 40 (47.62) |  |  |
| ≥150 | 245 (50.2) | 201 (49.75) | 44 (52.38) |  |  |
| CA19-9, n (%) |  |  |  | χ²=2.288 | 0.130 |
| <34 | 338 (69.26) | 274 (67.82) | 64 (76.19) |  |  |
| ≥34 | 150 (30.74) | 130 (32.18) | 20 (23.81) |  |  |

This table summarizes patient data on key clinically significant variables only. BMI, Body Mass Index; AGR, Albumin-to-globulin ratio; NLR, Neutrophil-to-lymphocyte ratio; PLR, Platelet-to-lymphocyte ratio; TBIL, Total bilirubin; ALT, Alanine aminotransferase; AST, Aspartate aminotransferase; ALP, Alkaline phosphatase; GGT, γ-glutamyl transpeptidase; CA19-9, Carbohydrate antigen19-9;
