# Supplementary material for: Correlation analysis and recurrence evaluation system for patients with recurrent hepatolithiasis: a multicentre retrospective study
Source: Front Digit Health. 2024 Nov 27;6:1510674. doi: 10.3389/fdgth.2024.1510674 (PMC11631919; doi:10.3389/fdgth.2024.1510674)
Supplement: Supplementary file 2 [file Table2.docx]

**Table 2.** The number of recurrent patients in k years

| In k years | 1 | 2 | 3 | 4 | 5 |
| --- | --- | --- | --- | --- | --- |
| Number | 44 | 108 | 126 | 132 | 135 |
