# Supplementary material for: Correlation analysis and recurrence evaluation system for patients with recurrent hepatolithiasis: a multicentre retrospective study
Source: Front Digit Health. 2024 Nov 27;6:1510674. doi: 10.3389/fdgth.2024.1510674 (PMC11631919; doi:10.3389/fdgth.2024.1510674)
Supplement: Supplementary file 3 [file Table3.docx]

**Table 3.** Area under the receiver operating characteristic curve (AUC) of each model at different time nodes

| Model | AUC within 1 year | AUC within 2 years | AUC within 3 years | AUC within 4 years | AUC within 5 years |
| --- | --- | --- | --- | --- | --- |
| XGBoost | 0.941 | 0.906 | 0.922 | 0.917 | 0.887 |
| LightGBM | 0.981 | 0.924 | 0.889 | 0.907 | 0.885 |
| RF | 0.903 | 0.825 | 0.852 | 0.849 | 0.774 |
| SVM | 0.900 | 0.856 | 0.836 | 0.843 | 0.832 |
| AdaBoost | 0.659 | 0.779 | 0.732 | 0.661 | 0.781 |
| NNW | 0.747 | 0.852 | 0.823 | 0.845 | 0.813 |
| DT | 0.469 | 0.650 | 0.674 | 0.636 | 0.542 |
| LR | 0.819 | 0.839 | 0.810 | 0.833 | 0.795 |
| KNN | 0.600 | 0.592 | 0.585 | 0.576 | 0.568 |

This table summarizes area under the receiver operating characteristic curve (AUC) of each model at different time nodes only. Additional data on optimal parameters and performance of each model is summarized in supplementary appendix. XGBoost, Extreme Gradient Boosting; LightGBM, Light Gradient-Boosting Machine; RF, random forest; SVM, support vector machine; AdaBoost, Adaptive Boosting; NNW, neural network; DT, decision tree; LR, logistic regression; KNN, K-nearest neighbour
